# Vapor-Phase Photocatalytic Overall Water Splitting Using Hybrid Methylammonium Copper and Lead Perovskites

**DOI:** 10.3390/nano10050960

**Published:** 2020-05-18

**Authors:** Teresa García, Rocío García-Aboal, Josep Albero, Pedro Atienzar, Hermenegildo García

**Affiliations:** Instituto Universitario de Tecnología Química CSIC-UPV, Universidad Politecnica de Valencia, Av. de los Naranjos s/n, 46022 Valencia, Spain; letuana_@hotmail.com (T.G.); rogarab@itq.upv.es (R.G.-A.); joalsan6@upvnet.upv.es (J.A.)

**Keywords:** hybrid perovskite, photocatalysis, water splitting, hydrogen production

## Abstract

Films or powders of hybrid methylammonium copper halide perovskite exhibit photocatalytic activity for overall water splitting in the vapor phase in the absence of any sacrificial agent, resulting in the generation of H_2_ and O_2_, reaching a maximum production rate of 6 μmol H_2_ × g cat^−1^h^−1^ efficiency. The photocatalytic activity depends on the composition, degreasing all inorganic Cs_2_CuCl_2_Br_2_ perovskite and other Cl/Br proportions in the methylammonium hybrids. XRD indicates that MA_2_CuCl_2_Br_2_ is stable under irradiation conditions in agreement with the linear H_2_ production with the irradiation time. Similar to copper analogue, hybrid methylammonium lead halide perovskites also promote the overall photocatalytic water splitting, but with four times less efficiency than the Cu analogues. The present results show that, although moisture is strongly detrimental to the photovoltaic applications of hybrid perovskites, it is still possible to use these materials as photocatalysts for processes requiring moisture due to the lack of relevance in the photocatalytic processes of interparticle charge migration.

## 1. Introduction

Hybrid organic inorganic perovskites are attracting currently considerable attention due to their performance in photovoltaic devices, where efficiencies above 23% [1] have been achieved in a very short time compared to the slower progress that has characterized the efficiency increase evolution in dye sensitised solar cells [2,3,4,5,6,7,8]. Hybrid perovskites offer a large flexibility for tuning their optoelectronic and other properties including absorption spectrum, bandgap, and band level energy by controlling their composition, average particle size, and film preparation process [9,10,11,12]. The high coefficient of visible light absorption characteristic of hybrid perovskites, together with their efficient charge separation and charge carrier mobility, are among the main features responsible for the performance of this type of materials in photovoltaic cells [13,14].

These fundamental photochemical processes are also common in many other applications beyond solar cells [15,16,17]. Specifically in photocatalysis, the energy of the photons is frequently converted into a charge separation state that is able to promote the oxidation and reduction of substrates by holes and electrons, respectively [18]. 

In spite the fact that inorganic perovskites, such as those containing Ti and Ta, are among the most efficient photocatalysts for hydrogen generation from water by solar light and the large current interest in hybrid perovskites, there have been no reports of the use of hybrid perovskites as photocatalyst for overall water splitting [19,20,21]. The main reason for this lack of studies of the use of hybrid perovskites as photocatalysts for hydrogen generation is the well-known lack of stability of hybrid perovskites in the presence of water, light and even moisture [22,23,24]. Although the negative influence of the humidity in the performance of photovoltaic devices based on hybrid lead perovskites is a well-known fact, there is still an incomplete understanding of the reasons. One of the proposals is that moisture hydrates the external surface of hybrid perovskite grains in the film, decreasing its electrical conductivity by disfavouring interparticle electron migration [25]. In other studies, it has been proposed that water reacts reversibly with MAPbI_3_ films through the initial formation of aqueous complexes that finally degrade the generation of PbI_2_ [26]. Major studies have claim that the degradation promoted by hydration lead to the release of CH_3_NH_2_ and HI as gases [27]. In any case, according to the proposed degradation mechanism, one water molecule could be sufficient to completely degrade the MAPbI_3_ film. Nevertheless, the degradation mechanism of hybrid lead halide is still under debate and further studies to understand the possible role of highly conductive PbI_2_ hydrate are still attracting considerable attention [28]. 

**Scheme 1:**
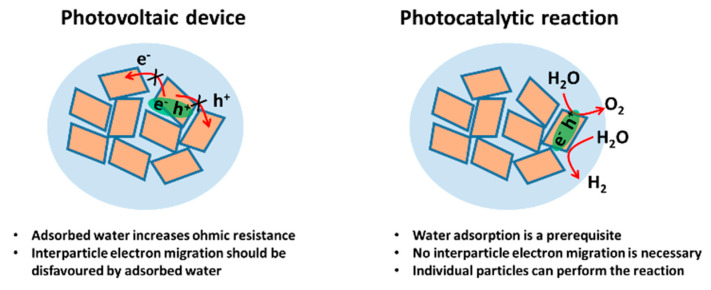
Cartoon illustrating the main differences between the operation mechanisms of a photovoltaic device and a photocatalytic system. These differences can serve to rationalize why the detrimental effect of H_2_O in solar cells could not preclude the occurrence of an efficient photocatalytic reaction.

In any case, even if the presence of moisture is strongly detrimental for photovoltaic devices, there could be still a possibility for efficient photocatalytic water splitting using hybrid metal halide if the photocatalytic reaction is sufficiently fast under the experimental conditions. In photocatalysis photogenerated electrons and holes do no need to migrate from particle to particle up to the external electrode because the reaction takes place just on the surface of each semiconductor particle where moisture should be reactive [26,29,30]. The main differences between the operation of a photovoltaic device and a photocatalytic reaction that will make feasible water splitting are summarized in Scheme 1. There are examples in the literature in which photocurrents are not observed, while the material exhibits photocatalytic activity [31]. Therefore, there is still a chance for observing highly efficient photocatalysis even though moisture plays a strong detrimental effect in photovoltaic devices [32].

Based on this consideration and aimed at providing information on the photocatalytic activity of hybrid perovskites, in the present study we report the photocatalytic activity for overall water splitting of lead-free copper-based hybrid methylammonium halide perovskite. The selection of this hybrid copper perovskite was made to avoid the use of toxic metals such as lead that in the present case is replaced by an Earth abundant first-row transition metal and also trying to study a material whose photochemical properties are less influenced by humidity, particularly when chloride is present in its composition [33,34,35]. The synthesis, structure, and energy levels of a series of MA_2_CuCl_x_Br_4-x_ hybrid perovskites (MA: methylammonium) have been recently reported, including film preparation and photovoltaic activity of the material [36]. In the present study, we have selected MA_2_CuCl_2_Br_2_ and MA_2_CuCl_0.5_Br_3.5_ to determine their ability for photocatalytic overall water splitting upon simulated sunlight irradiation in the gas phase of films of these materials [36]. A comparison with the photoresponse of hybrid lead perovskite is also provided.

## 2. Materials and Methods 

### 2.1. Synthesis of MA_2_CuCl_x_Br_4-x_ Perovskite Powders

Preparation of the hybrid perovskites was performed by crystallization of a mixture of MACl and MABr in slight excess with respect to a mixture of CuCl_2_ and CuBr_2_, as previously reported in the literature [36]. Powders of these copper perovskites were prepared by crystallization from ethanol solutions, employing 1.34 g of CuBr_2_ (copper bromide, 99% Sigma-Aldrich, Darmstadt, HE, Germany), and 0.972 g of MACl (methylammonium chloride, 99% Sigma-Aldrich, Darmstadt, HE, Germany) for MA_2_CuCl_2_Br_2_.

For the other mixed halide copper perovskites, the precursors were mixed in 100 mL of EtOH, using 1.2 equiv. of organic precursor with respect to a mixture of copper salts as previous reported in the literature [36]. Then, the solutions were stirred for 2 h at 60 °C. Crystal growth was carried out by keeping overnight the ethanolic solutions in a freezer. The crystals were recovered by filtration, dried overnight at 60° C in a vacuum oven, and finally stored in glovebox.

### 2.2. Films Fabrication

DMF solutions of MA_2_CuCl_2_Br_2_ (2 M) were prepared by dissolving powders of this perovskite at room temperature. After that, thin films of catalyst were made depositing 25 µL of the DMF solution in a 2 × 2 cm^2^ quartz square by drop casting and annealing on a hot plate at 70 °C for 1 h. After film preparation, the specimens were kept in a glovebox.

### 2.3. Materials Characterization

XRD patterns of the powders and thin films were recorded on a Philips (Amsterdam, The Netherlands) X’PERT diffractometer that was equipped with a proportional detector and a secondary graphite monochromator. The data were collected step wise over the range 2θ = 2–20°, at steps of 0.02°, an accumulation time of 20 s/step, using the Cu Kα radiation (λ = 1.54178 Å). 

UV–Vis optical spectroscopy of the perovskite films was carried out using a Cary 5G spectrophotometer (Santa Clara, CA, USA) and CaSO_4_ as reference.

Field-emission scanning electron microscopy (FESEM) images were recorded with a Zeiss Ultra 55 field FESEM apparatus (Atlanta, GA, USA).

### 2.4. Transient Absorption Spectroscopy (TAS)

Transient absorption experiments were recorded using a home-built system as reported before [37]. The samples were excited with a Nd:YAD laser at 532 nm and 1 Hz repetition rate; <20 ns pulse duration. The resulting photoinduced changes in optical density were monitored by employing a 150 W Tungsten lamp, with 20 nm bandwidth monochromators before and after sampling, a home-built photodiode-based detection system, and a MDO-3034 Tecktronic oscilloscope.

### 2.5. Photocatalytic Measurements

The photoreactor to evaluate the photocatalytic activity of hybrid perovskites consists in a homemade stain steel body with a top quartz window with a truncated conical shape. The total capacity of the photoreactor is 120 mL. The temperature and pressure inside the photoreactor were measured by an internal thermocouple and a manometer, respectively.

Supplementary Figure S1 shows a photograph of the photoreactor used, indicating location of the thermocouple and the distribution of the catalyst and the reagents inside the reactor. The photoreactor was located 20 cm below the solar simulator collimated beam and the maximum temperature of gas mixture was 40 °C. Argon was introduced into the reactor to avoid the presence of oxygen and 1 mL of water was used as reagent. The water drops was placed in a separated container of 10 mL to keep constant the ambient humidity. The amount of photocatalyst was 10 mg. The evolution of hydrogen and oxygen was determined by direct connection of the photoreactor to an Agilent 490 Micro GC (Santa Clara, CA, USA) (Molsieve 5A column with Ar as carrier gas). The light source was a 150 W lamp. Simulated sunlight irradiations were carried out using a solar simulator (Sun 2000 ABET technologies 1000 W/m^2^) (Milford, CT, USA) equipped with an AM 1.5G filter. Irradiation were performed in triplicate using samples prepared independently, the maximum error of the measurement was 15%. Analysis of the possible presence of H_2_O_2_ was carried out by washing the photocatalyst and photoreactor with Milli-Q water and proceeding with colorimetric test of H_2_O_2_. No evidence of the formation of any H_2_O_2_ was obtained (see Figure S2).

## 3. Results and Discussion

### 3.1. Photocatalyst Characterization

**Figure 1:**
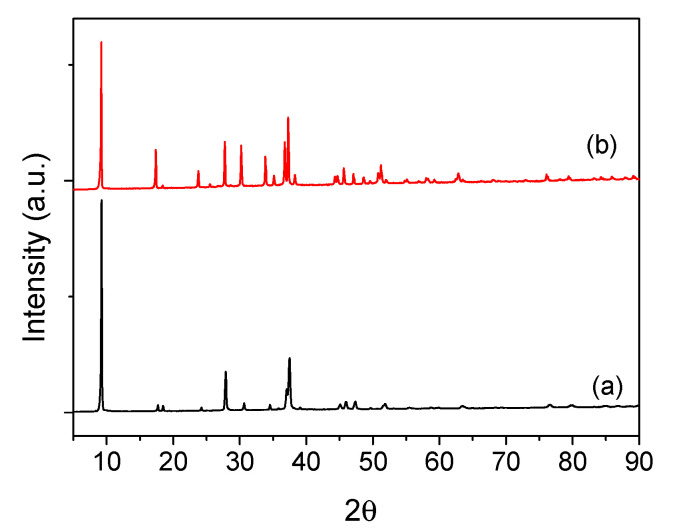
X-ray patterns of as-synthesized hybrid copper perovskites powders: (**a**) MA_2_CuCl_2_Br_2_ and (**b**) MA_2_CuCl_0.5_Br_3.5_.

The resulting powders were characterized by XRD (Figure 1) where the diffraction peaks expected for the hybrid MA_2_CuX_4_ perovskites were recorded, agreeing with the data of the original article in which the synthesis of these copper perovskites was reported [36]. 

**Figure 2:**
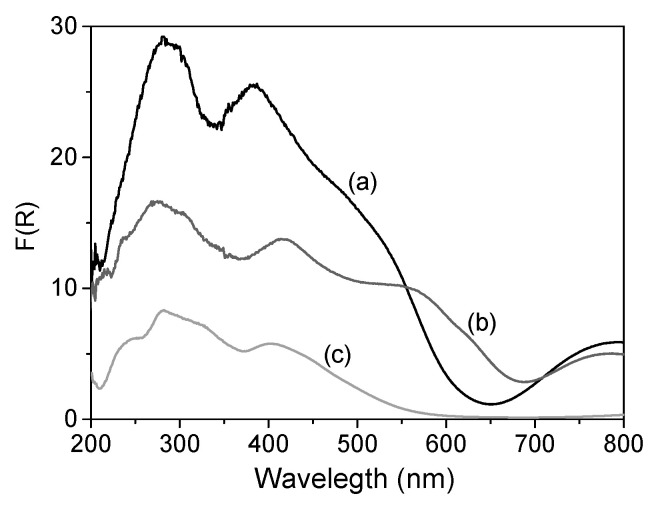
Diffuse reflectance UV–Vis absorption spectra (plotted on the Kubelka-Munk function of the reflectance, F(R) of hybrid copper perovskite powders: (**a**) MA_2_CuCl_2_Br_2_, (**b**) MA_2_CuCl_0.5_Br_3.5_ and (**c**) Cs_2_CuCl_2_Br_2_.

Optical spectroscopy shows the Cl^-^ or Br^-^ to Cu electronic transitions at λ_max_ 300 and 400 nm reaching up to 600 nm (MA_2_CuCl_2_Br_2_) or 680 nm (MA_2_CuCl_0.5_Br_3.5_) in accordance with the literature [36]. Intraband d-d electronic transitions on Cu^2+^ ions were also observed in the 700–1000 nm region as reported (Figure 2).

**Figure 3:**
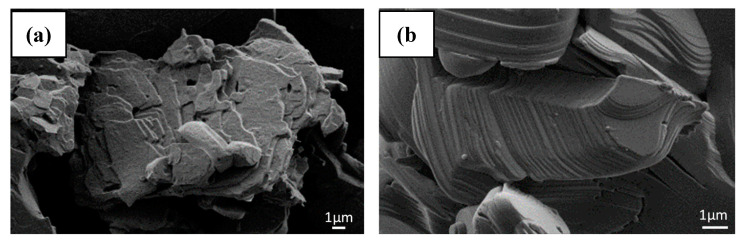
FESEM images of MA_2_CuCl_2_Br_2_. Powder sample 4000× (**a**) and film sample 8000× (**b**).

The morphology of the hybrid copper perovskites and the particle size can be determined by FESEM images. Figure 3 shows that the hybrid copper perovskites are constituted by very large particles between 40 and 100 µm formed by stacking of platelets of 50–100 nm thickness and much larger lateral area.

As previously commented in the introduction, formation of aqua-complex seems to be the first step in the water promoted degradation of hybrid lead perovskite resulting in the deterioration of its photovoltaics response [26,27,28]. To addresses the possible formation of an aqua complex in the case of MA_2_CuCl_2_Br_2_ the XPS spectra of the solid in the absence of humidity and after exposure to H_2_O in a pre-chamber of the instrument, immediately prior to record the spectra were compared. XPS is a surface technique that proves the external source of the film in contact with H_2_O and changes in the XPS Cu2p_3/2_ peak should report on the possible H_2_O coordination to Cu^2+^ or hydrolysis at the methylammonium group by changes in the binding energy and width of the peak. As shown in Supplementary Figures S3 and S4 a perfect coincidence of the Cu2p_3/2_ and N1s peaks were recorded for the fresh and H_2_O exposed MA_2_CuCl_2_Br_2_ samples, indicating that formation of an aqua complex in where H_2_O coordinates to Cu^2+^ replacing any of the halides coordinating to Cu^2+^ or hydrolysis of methyl ammonium does not occur in the presence of H_2_O, in spite of detection of O1s peak in the case of H_2_O exposed sample (see Figure S5). These XPS measurements suggest that H_2_O physisorbed on the surface of MA_2_CuCl_2_Br_2_ without affecting the coordination sphere of the Cu^2+^ ions. These coincidence upon prolonged (three weeks irradiation) also rules out that oxygen involved in the photocatalytic water splitting is consumed in the decomposition of the photocatalyst. Further details on the stability of MA_2_CuCl_2_Br_2_ upon prolonged exposure to moisture under photocatalytic conditions are provide bellow.

### 3.2. Photocatalytic Measurements

**Figure 4:**
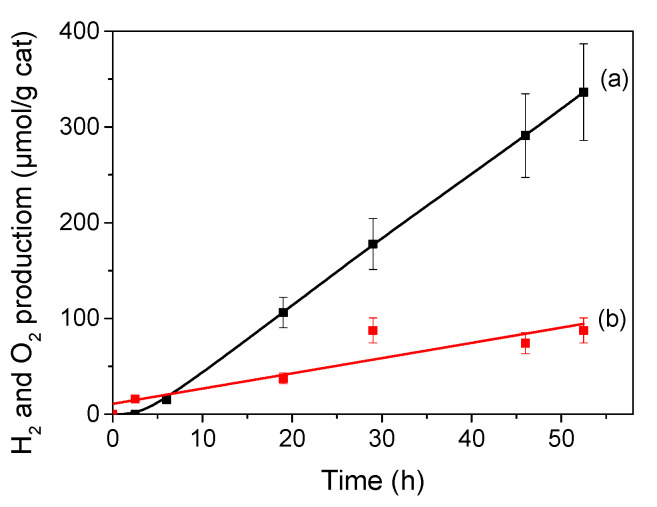
Temporal hydrogen (**a**) and oxygen (**b**) evolution upon simulated sunlight irradiation (100 mW cm^−2^) of MA_2_CuCl_2_Br_2_ films exposed to a H_2_O-satured atmosphere at 1 atm and 40 °C.

Photocatalytic experiments were carried out by illuminating with a solar simulator (1000 W/m^2^) powders or films of the hybrid perovskites at temperatures below 40 °C in a sealed photoreactor saturated with H_2_O vapor at atmospheric pressure that corresponds to a relative humidity of 100% (dewpoint). Supplementary Figure S1 shows a photograph of the setup used in these experiments. Since MA_2_CuCl_2_Br_2_ dissolve in water, it is not possible perform the photocatalytic water splitting by suspending MA_2_CuCl_2_Br_2_ in water. In contrast, it was found that exposure of MA_2_CuCl_2_Br_2_ to H_2_O vapour was sufficient to observe the photocatalytic reaction. In addition having the solid catalyst deposited on a surface can allow it to continuous flow photocatalytic experiments recirculating the gas phase. Hydrogen and oxygen evolution was quantified by a microGC connected in line with the photoreactor using a thermoconductivity detector and argon as carrier gas. Under these conditions, evolution of hydrogen and oxygen as a function of irradiation time was observed. Figure 4 shows a time evolution plot for a representative experiment. The apparent induction period for hydrogen evolution observed in Figure 4 is likely be due to some absorption phenomena, detection limit, and filling of the reactor volume.

Control experiments in the absence of water vapor allowed detecting only negligible amounts of hydrogen (less 1% of the hydrogen measured when water vapors were present). Also oxygen was undetectable under this condition in the absence of water saturation. This control experiment indicates that water is needed to observe the generation of hydrogen and oxygen. The temporal evolution of hydrogen follows a linear relationship with time, supporting the stability of the material as photocatalyst under the irradiation conditions. A maximum amount of hydrogen of 340 µmol × *g* cat^−1^ was achieved at 50 h irradiation time. Although the temporal evolution of oxygen did not follow strictly the expected stoichiometry for overall water splitting because initial and final irradiation times were somewhat higher and lower, respectively, than expected, oxygen amounts were quantified, and the generation of oxygen was clearly measured. This lack of linearity of oxygen production and its apparent constant concentration beyond 24 h can probably reflect a consumption of some amount of photogenerated oxygen inside the photoreactor. To asses or rule out the possibility of O_2_ consummation by formation of H_2_O_2_, analysis of the photocatalyst and photoreactor aim at detection of peroxides with Ti = O^2+^ was carried out. No evidence of the presence of peroxides was obtained. Analysis of the XPS Cu2p_3/2_ peak of the MA_2_CuCl_2_Br_2_ film after extensive irradiation in the presence of moisture (3 weeks) this not allow to detect significant variations in the binding energy and position of the peak respect to the fresh sample (Supplementary Figure S3). XPS data on the surface copper conclusively indicate that the photocatalyst is stable under the reaction conditions.

Overall water splitting typically requires co-catalyst in order to achieve measurable efficiencies. We speculated that in the present case overall water splitting activity could be favour by some CuO impurity acting as co-catalyst. Aim at clarify this issue we prepared an additional film in which CuO nanoparticles were deposited at 1% loading on MA2CuCl_2_Br_2_ by impregnation by DMF. However, no enhancement of the photocatalytic activity for this Cu modified film was observed with respect to the MA_2_CuCl_2_Br_2_ film lacking CuO nanoparticles.

The influence of the composition on the photocatalytic activity for overall water splitting under simulated sun light irradiation was addressed by performing analogous measurements using MA_2_CuCl_0.5_Br_3.5_ as photocatalyst. As commented earlier, hybrid perovskite based exclusively in bromide is known to be more deliquescent than the mixed Cl/Br material. Therefore, the presence of Cl^-^ should increase the stability of the photoresponse of the material in the presence of moisture. The measurements indicate that MA_2_CuCl_0.5_Br_3.5_ also exhibit photocatalytic activity for overall water splitting in the absence of the sacrificial agents, but with an efficiency about one half that of the MA_2_CuCl_2_Br_2_ (Supplementary Figure S6). Furthermore, prolonged irradiation time indicates some photocatalyst deactivation for MA_2_CuCl_0.5_Br_3.5_ as shown by the decrease hydrogen and oxygen production rate over the time.

For overall water splitting to occur, electrons in the conduction band (CB) and holes in the valence band (VB) must have adequate energy. Scheme S1 shows the reported potentials of VB maximum and CB minimum for MA_2_CuCl_2_Br_2_. It is noted that according of these reported values not oxygen generation should be possible for MA_2_CuCl_2_Br_2_, since it would require a potential about 5.4 eV [38,39]. However several possibilities can be considered to rationalize why oxygen evolves in the simulated sun light irradiation of MA_2_CuCl_2_Br_2_ in contact with H_2_O vapor. Thus, it has been reported that the Fermi level (E_F_) and work function (W_F_) of a given material can shift as much as 0.6 eV depending on the structure and surface morphology of the particles, exposure to the ambient and even the history of sample preparation [40]. For this reason, we proceeded to measure experimentally the VB energy maximum by XPS of our MA_2_CuCl_2_Br_2_ sample freshly and immediately after exposure to moisture in the XPS prechamber. The results are presented in Figure S7. The VB energy maximum was determined by extrapolation of the lineal slope of the peak intensity versus energy. As it can be seen in this figure, minor variations towards higher energy of 0.1 eV were determined upon moisture exposure from the initial 5.6 eV for the fresh sample. However, the necessary vacuum requirement of XPS could make that real value of VB maximum in the photocatalytic experiment could be even higher due to the higher H_2_O pressure. There are precedents in the literature showing a shift in the VB energy depending on the presence of water. Whatever the reason, generation of oxygen from H_2_O in the absence of sacrificial electron donors opens new avenues for the development of visible light responsive photocatalyst based on hybrid perovskites.

It is known that in overall water splitting, oxygen generation is the rate determining step since it requires four holes for the formation of each O_2_ molecule [41]. Accordingly, a general practice to determine the maximum hydrogen production rate is to perform photocatalytic experiments also in the presence of sacrificial electron donors, amines being among the preferred electron donors [42]. In these experiments, an excess of sacrificial agents is generally added in proportions as high as 30% with respect to water [43]. Unfortunately, triethylamine in excess produces the complete decomposition of MA_2_CuCl_2_Br_2_ and this type of experiments of photocatalytic hydrogen generation could not be done under optimal conditions requiring an excess of amine as sacrificial agent. Nevertheless, measurements of hydrogen evolution using MA_2_CuCl_2_Br_2_ as photocatalyst were done adding a minimal amount of methylamine. It was observed that the rate of hydrogen generation at 4 h irradiation time increases by a factor of about two, from 3.85 to 8.85 µmol H_2_/g. cat. when some amine is present. This influence of the presence of methylamine lends further support to the general assumption that also in the case of MA_2_CuCl_2_Br_2_ the photocatalytic activity for hydrogen generation is limited by the slower O_2_ evolution. 

**Figure 5:**
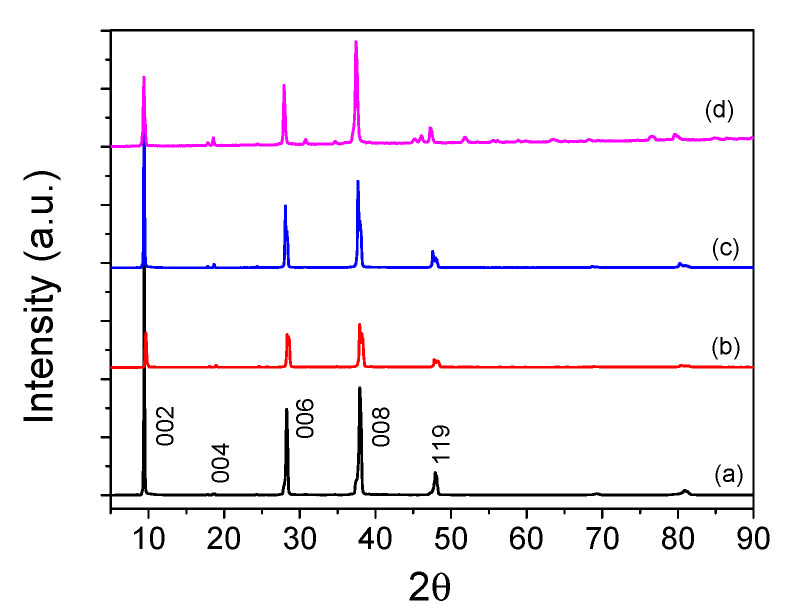
XRD patterns of MA_2_CuCl_2_Br_2_ perovskite powders upon irradiation with a solar simulator (1000 W/m^2^): (**a**) fresh sample, (**b**) after 24 h, (**c**) 48 h and (**d**) three weeks reaction. The relatively minor changes in the relative peak intensity and FWH peak are due to unavailable variation in the packing of the solid and surface levelling when filing the cell holder.

As commented before, photocatalytic experiments were performed using thin films of hybrid copper perovskite on quartz substrate prepared by casting the solid photocatalyst suspended in DMF 2M followed by slow evaporation of the solvent at 70 °C. An alternative procedure consists in performing the photocatalytic experiments with dry powders of the hybrid copper perovskite. Also, under these conditions, the exposure of the powders in thin bed (10 mg) to UV-Vis light in the presence of water vapour allows for the detection of the evolution of significant amounts of hydrogen and oxygen (see Figure 4). However, in this case, due to the use of an excess mass of the solid material with respect to the experiments in where thin films were used, much lower specific evolution rates were measured. However, these experiments with dry powders complement the previous XPS data on MA_2_CuCl_2_Br_2_ films, confirming the photochemical stability of the hybrid copper perovskite under photocatalytic conditions upon exposure for extended irradiation times. The use of powder MA_2_CuCl_2_Br_2_ samples allows monitoring the XRD pattern as a function of the irradiation time. Minor changes in the XRD patterns of copper perovskite consisting in a broadening of the peak width (FWHP from 0.26 to 0.42 of the 006 peak) and a decrease of the 002 peak intensity (from 3 to 1 I_002_/I_006_) were observed. The results are presented in Figure 5. These changes were reversible in some degree and the same sample recover partially the intensity of the 002 peak and the peaks became narrower upon standing the material under the photocatalytic conditions without any treatment. This reversibility of XRD pattern changes in the presence of moisture have been reported for MAPbI_3_ films and single crystals upon exposure to moisture and purpose to be due to hydration of the hybrid lead perovskite. Further experimental evidence support the stability of MA_2_CuCl_2_Br_2_ under prolonged photocatalytic experiments was obtained by XPS (Supplementary Figure S3). Comparison of the high resolution XPS Cu2p_3/2_ peak of the fresh and hydrated material with that after three weeks of continuous irradiation does not show any difference and the peaks were coincident. All these data support the stability of the material under the conditions where hydrogen and oxygen are formed. The conclusions based in XRD was mainly confirmed by XPS data of the sample after been used as photocatalyst by analysing the Cu2p_3/2_ peak. However, deconvolution of the experimental XPS Cu2p_3/2_ peak on the sample used of photocatalyst suggest, although the peak is almost unchanged, the presence of a minor component (about 20%) of a contribution of an additional component (binding energy 530 eV) that could correspond to the formation of a minor proportion of CuO on the outer moisture surface of the photocatalyst.

Besides hybrid copper perovskites and considering the large diversity of hybrid perovskites that have been prepared for evaluation in photovoltaic cells [44], we also screened the photocatalytic activity of some related hybrid perovskites. Specifically, to determine the role of the organic MA^+^ ion a full inorganic copper perovskite in where the MA^+^ was replaced by Cs^+^ was also prepared and tested as photocatalyst for overall H_2_O splitting. It is well known in the state of the art that the composition of perovskites exerts a large influence in the optoelectronic properties as well as in the efficiency of light-to-current conversion [45]. With these precedents in mind, it was expected that the replacement of MA^+^ by Cs^+^ should result in a change in the photocatalytic activity. The activity measured for Cs_2_CuCl_2_Br_2_ in the overall water splitting is provided in Table 1.

As shown in Table 1, although Cs_2_CuCl_2_Br_2_ also exhibits photocatalytic activity for overall water splitting in the absence of any sacrificial agent, the activity determined for this material was significantly smaller than that achieved using MA_2_CuCl_2_Br_2_ that was the best photocatalyst of the series (see Table 1). This lower activity of Cs^+^ perovskite with respect to that of MA^+^ hybrid perovskite has also been observed with regard to the photovoltaic efficiency in solar cell devices and has been attributed to the combination of several factors, the most important being the increase in the band gap of the Cs^+^ material and possible structural changes. As it can be seen in the Figure 2 also in the present case Cs_2_CuCl_2_Br_2_ exhibits larger bandgap than the analogous MA^+^ hybrid perovskite, meaning that less photons in the visible range are absorbed in Cs_2_CuCl_2_Br_2_ compared to MA_2_CuCl_2_Br_2_ and this should result in lower photocatalytic activity. Concerning the spectral photoresponses, it can be assumed that they will follow a similar trend to the photocurrent performed for similar perovskites on photovoltaic devices, where the main photoresponse is observed for wavelengths shorter than 650 nm [36].

Besides copper perovskites, it was of interest to determine the possible photocatalytic efficiency of hybrid lead halide perovskites, since these materials are the ones that exhibit the highest light-to-current conversion efficiency in photovoltaic devices [36]. Although the photocatalytic activity varied depending on the halide, being particularly active the one having Br and I, their efficiency was lower than those of copper perovskites.

To put the data shown in Table 1 in a broader context it should be noted that the STH data refer to overall water splitting in the absence of any noble metal co-catalyst. Values for other overall water splitting co-catalyst under comparable conditions (solar light, absence of sacrificial, absence of co-catalyst) give similar or lower STH values [46,47]. It should also be noted that, considering the flexibility in the synthesis of hybrid metal halide perovskites, other related perovskites could even exhibit higher efficiency.

As mentioned in the introduction, hybrid lead perovskites are influenced by the presence of moisture, but we have previously commented that this negative influence with respect to the photovoltaic efficiency due to the increase of the resistance of the interparticle migration of charge carriers should not be necessarily much detrimental for the photocatalytic activity. Interparticle charge migration is not an elementary process occurring in photocatalysis. Also the experimental conditions employed in the overall H_2_O splitting only expose hybrid perovskites to water vapor and not to liquid water. Photocatalytic experiments using hybrid lead perovskite established also for this material as film on glass substrates their ability to generate hydrogen and oxygen upon irradiation with simulated solar light. The efficiency of the photocatalytic process also depends on the composition of the lead perovskite and among the materials tested, the most efficient one was MAPbBrI_2_ that was somewhat more efficient than the other MA^+^ lead perovskites tested, all of them exhibiting very similar performance. It is proposed that the higher activity of Br/I can derive from the band gap alignment in this material with respect to the potentials required for hydrogen generation and oxygen evolution and from its relatively narrow band gap (1.8 eV) that allows absorption of more photons in the visible range [13,40,48]. Other compositions of perovskites either have the absorption onset shifted towards the UV region or have too narrow bandgap to promote efficient overall water splitting. In any case, it seems that the efficiency of lead halide perovskite is about four times lower than that presented in Figure 4 for hybrid copper perovskite.

### 3.3. Transient Absorption Spectra of MA_2_CuCl_2_Br_2_

**Figure 6:**
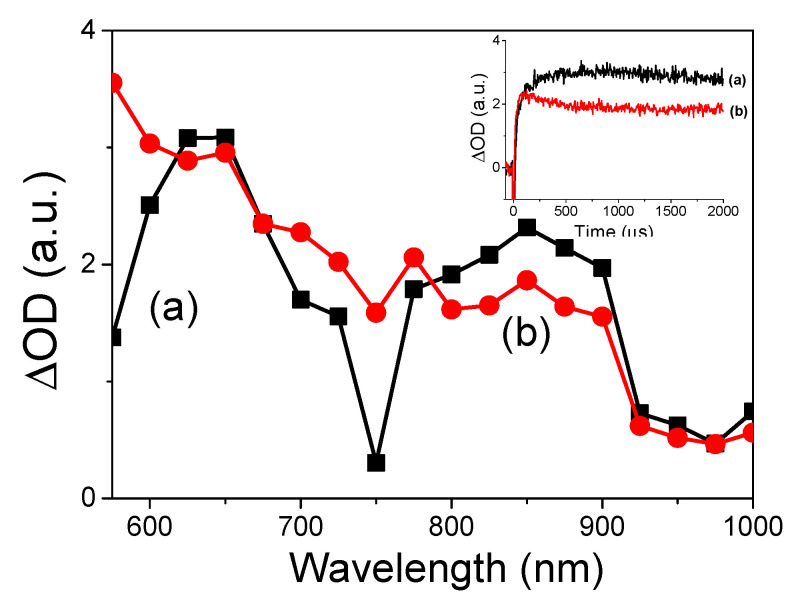
Transient spectra recorded at 100 (**a**) and 1000 μs (**b**) after 532 nm laser excitation of a N2 purged film of MA_2_CuCl_2_Br_2_. The inset shows the signal profiles monitored at 580 nm (**a**) and 850 nm (**b**).

In order to gain some evidence in support of the photogeneration of electrons and holes upon light absorption in MA_2_CuCl_2_Br_2,_ a transient absorption study using 532 nm as excitation wavelength was carried out. The transient absorption spectra recorded for MA_2_CuCl_2_Br_2_ at 100 and 1000 μs after laser excitation is presented in Figure 6. As it can be seen there, at short time delays after the laser pulse, the transient absorption consisted in two absorptions bands at λ_max_ about 640 and 850 nm. At longer time delays the transient absorption at shorter wavelengths (λ < 600 nm) increases and the presence 640 nm band became less defined. This growth in the shorter wavelength region of the spectrum is clearly observed monitoring the transient signal at this region (inset of Figure 6). As it can be seen, the temporal profile of the signal at 580 nm grows in the first 500 μs and then becomes stationary during the longest time-window available in our nanosecond laser systems (2000 μs). It is proposed that this growth kinetics in the first 500 μs corresponds to kinetics of delocalization of electrons and holes within the MA_2_CuCl_2_Br_2_.

**Figure 7:**
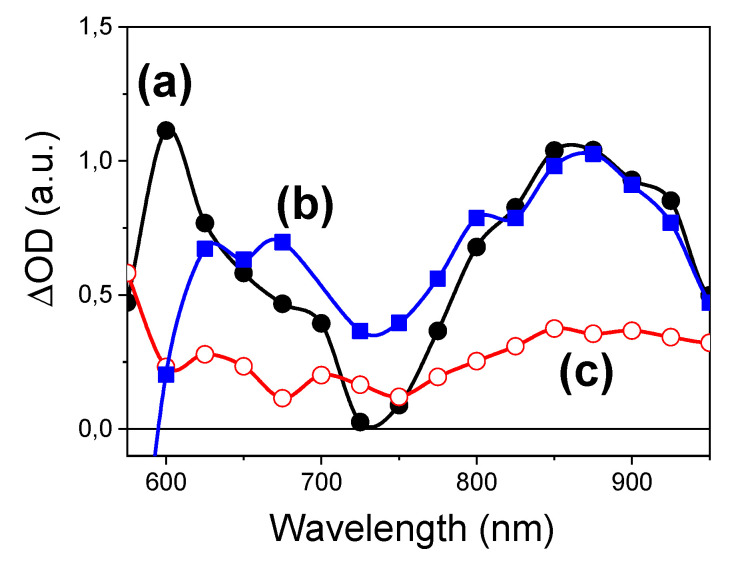
Transient spectra recorded at 100 μs after 532 nm laser excitation of a N_2_ purged film of MA_2_CuCl_2_Br_2_ (**a**) and after exposure to methanol (**b**) or oxygen (**c**).

To learn about the nature of these two absorption bands at 640 and 850 nm and the corresponding transient species, quenching experiments by methanol (electron donor quencher) and oxygen (electron acceptor quencher) were carried out. It was observed that methanol quenches the signal at 640 nm while it does not affect to the 850 nm band. This behaviour indicates that the two bands should correspond to two different species, the one at 640 nm corresponding to species with character of positive holes quenchable by methanol as electron donor, while this character of positive holes is not exhibited by the 850 nm band. Oxygen quenching leads to the complete disappearance of the transient signal in the submillisecond time scale at any wavelength. Since the 650 nm band was attributed based on methanol quenching to species with positive character, that in principle should not be quenchable by oxygen, it is more likely that besides oxygen as electron acceptor, it acts also as electron spin quencher, deactivating in the present case precursors of the charge separated state with triplet character. Figure 7 shows representative transient signals to summarize the results of the methanol and oxygen quenching of the MA_2_CuCl_2_Br_2_ photogenerated transients.

**Figure 8:**
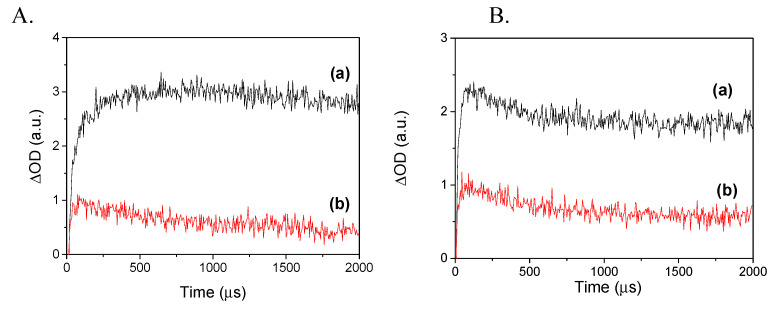
(**A**) Signal decay monitored at 640 nm before (**a**) and after water vapor exposure (**b**). (**B**) Signal decay monitored at 850 nm before (**a**) and after water vapor exposure (**b**).

After having addressed MA_2_CuCl_2_Br_2_ stability to moisture and proved that the transients detected in the μs time regime react with H_2_O, quenching studies of MA_2_CuCl_2_Br_2_ film transients upon excitation at 532 nm by H_2_O-saturated N_2_ were carried out. Figure 8 presents the main changes in the signal temporal profile monitored at 640 and 850 nm due to the presence of water vapour. It should be noted that both wavelengths undergo a clear quenching of the signal due to the presence of H_2_O, in contrast with the previously commented results for methanol that is exclusively a hole quencher. These temporal profiles provide a sound evidence of the reactivity of the charge separation state generated on MA_2_CuCl_2_Br_2_ with H_2_O. However, it should be noted that the presence of H_2_O at longer exposure times may cause a deterioration of the MA_2_CuCl_2_Br_2_ film response, and for this reason, H_2_O quenching experiments were performed exclusively with fresh samples.

**Scheme 2:**
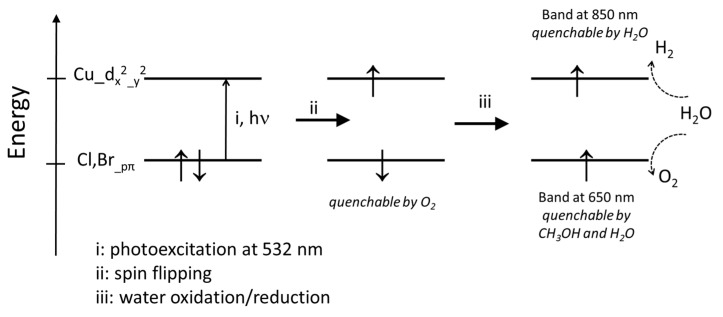
Mechanistic proposal to rationalize the transient absorption spectroscopy study in the photoexcitation of MA_2_CuCl_2_Br_2_.

The previous results, and particularly the quenching of the 640 and 850 nm bands by H_2_O suggest that these transient signals correspond to a charge separation state and that water can quench both sites with hole and electron character, this being compatible with the observation of overall water splitting. Since Cortecchia et al. have assigned the 532 nm absorption to the electronic transition from Cl,Br_pπ electrons to Cu_d_x_^2^__y_^2^, it is proposed that holes are localized at Cl,Br_pπ orbitals and they should be responsible for the 640 nm band. Electrons in the conduction band, on the other hand, will have a predominant location in Cu orbitals compatible with the long wavelength of the 850 nm band in the region typically assigned to Cu electronic transitions. Scheme 2 illustrates our proposal.

## 4. Conclusions

In the present work, we have reported that hybrid perovskites have photocatalytic properties for overall water splitting in the absence of sacrificial agents under simulated solar light irradiation. Among the series, the studied copper methylammonium halide perovskites have been found to be the most efficient ones, reaching a specific hydrogen generation for MA_2_CuCl_2_Br_2_ of 6 µmol of hydrogen per gram of catalyst/h under optimal conditions in where thin films of this hybrid copper perovskite are exposed to water vapour at temperature about 40 °C. The material appears to be stable under photocatalytic conditions according to XRD measurements that do not revealed changes in the crystallinity of powdered materials and also based on the linear temporal evolution of the hydrogen production. This photocatalytic activity seems to be general also for other hybrid perovskites, particularly those based on lead, although their efficiency is lower than that determined for MA_2_CuCl_2_Br_2_. Considering the large variety of hybrid perovskites that are currently being prepared for their screening in photovoltaic devices, the present study opens the way for their evaluation also as photocatalysts, taking advantage of the narrow band gap and tunability of the optoelectronic properties that these hybrid perovskites can offer. The main limitation appears to be stability under moisture, but this limitation can be overcome by performing photocatalytic experiments in the gas phase.

## Figures and Tables

**Table 1:** H_2_ production data at 24 h of reaction for different types of perovskites *.

Photocatalyst	µmol H_2_/gcat	AQY ^a^ (%)	TON ^c^
MA_2_CuCl_2_Br_2_	141.21	0.7153 (0.73) ^b^	5.06 (12.05) ^d^
MA_2_CuCl_0.5_Br_3.5_	94.23	0.4773	4.00
Cs_2_CuCl_2_Br_2_	9.03	0.0457	0.51
MAPbBr_2_	5.16	0.0261	0.30
MAPbClI_2_	2.89	0.0146	0.15
MAPbI_3_	2.69	0.0136	0.17
MAPbCl_3_	2.54	0.0129	0.09
MAPbBr_3_	2.11	0.0107	0.10

* Reaction conditions: photocatalyst 10 mg powder and irradiation with a solar simulator (1000 Wm^−2^). ^a^ Apparent Quantum Efficiency (AQY) calculated from the equation: AQY = 2·number of produced hydrogen molecules/number of incident photons. ^b^ Apparent Quantum Efficiency for oxygen production. ^c^ TON: turnover number calculated at 24 h. ^d^ TON calculated at 52 h.

## Supplementary Materials

The following are available online at https://www.mdpi.com/2079-4991/10/5/960/s1, Figure S1: Photocatalytic system, Figure S2: Colorimetric titration by titanyl on MA_2_CuCl_2_Br_2_, Figures S3, S4 and S5: XPS analysis of MA_2_CuCl_2_Br_2_, Figure S6: H_2_ generation of hybrid lead halide perovskites, Scheme S1: Energy Level positions, Figure S7: VB determination of MA_2_CuCl_2_Br_2,_ Figure S8: Temporal evolution of H_2_ generation for MA_2_CuCl_2_Br_2_ perovskite, Figure S9: Temporal evolution of H_2_ generation for MA_2_CuCl_2_Br_2_ and MA_2_CuCl_0.5_Br_3.5_ perovskites, Figure S10: Evolution of the optical absorption spectrum of the MA_2_CuCl_2_Br_2_ upon laser irradiation.
