# Supplementary material for: Vapor-Phase Photocatalytic Overall Water Splitting Using Hybrid Methylammonium Copper and Lead Perovskites
Source: Nanomaterials (Basel). 2020 May 18;10(5):960. doi: 10.3390/nano10050960 (PMC7279556; doi:10.3390/nano10050960)
Supplement: Supplementary file 1 [file nanomaterials-10-00960-s001.pdf]

Supplementary Material

Vapor-Phase Photocatalytic Overall Water Splitting Using Hybrid Methylammonium Copper and Lead Perovskites

Teresa García, Rocío García-Aboal, Josep Albero, Pedro Atienzar * and Hermenegildo García *

Instituto Universitario de Tecnología Química CSIC-UPV, Universidad Politecnica de Valencia, Av. de los Naranjos s/n, 46022 Valencia, Spain; letuana_@hotmail.com (T.G.); rogarab@itq.upv.es (R.G.A.); joalsan6@upvnet.upv.es (J.A.)
* Correspondence: pedatcor@itq.upv.es (P.A.); hgarcia@qim.upv.es (H.G.)

A)

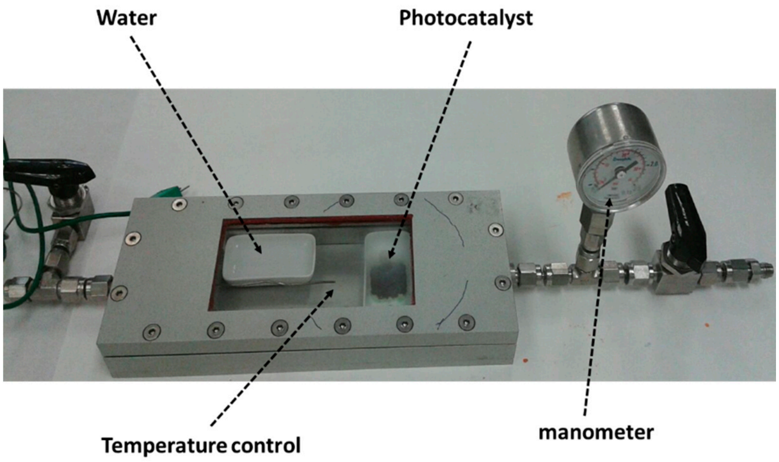

B)

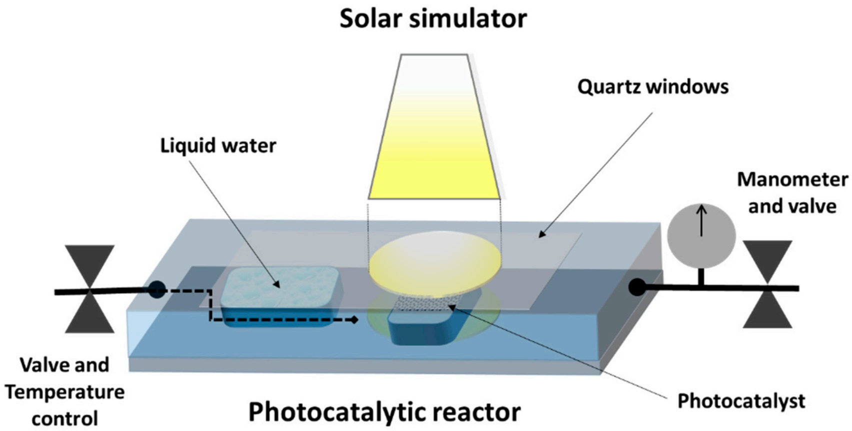

Figure S1. A) Home-made reactor employed for the photocatalytic activity measurements of the hybrid perovskites. Note that liquid water, providing the vapour for the photocatalytic

reaction is in a separate container close to the hybrid metal halide photocatalyst. B) Representation of the photocatalytic system during the reaction process.

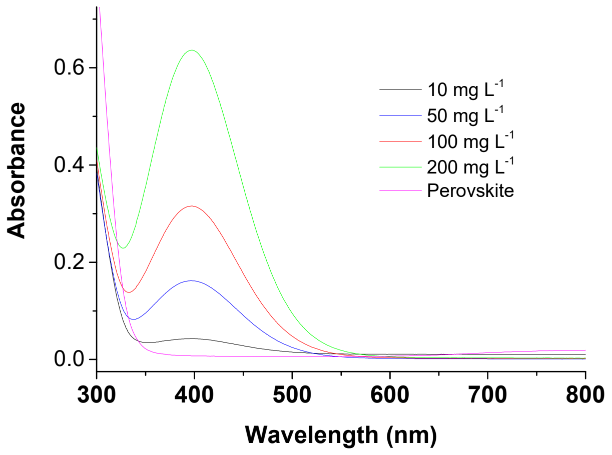

Figure S2. Colorimetric titration by titanyl on $MA_2CuCl_2Br_2$ to determine the H_2O_2 formation.[42]

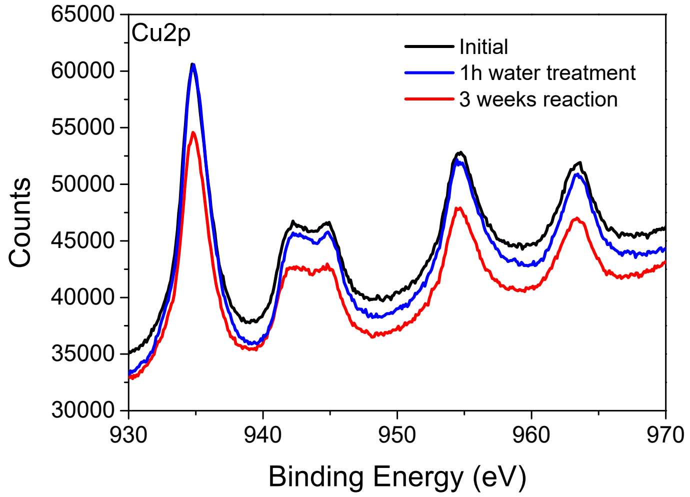

Figure S3. XPS analysis of $MA_2CuCl_2Br_2$ measured on a fresh sample, exposed to 1 hour water atmosphere (quickly measured) and after 3 weeks reaction.

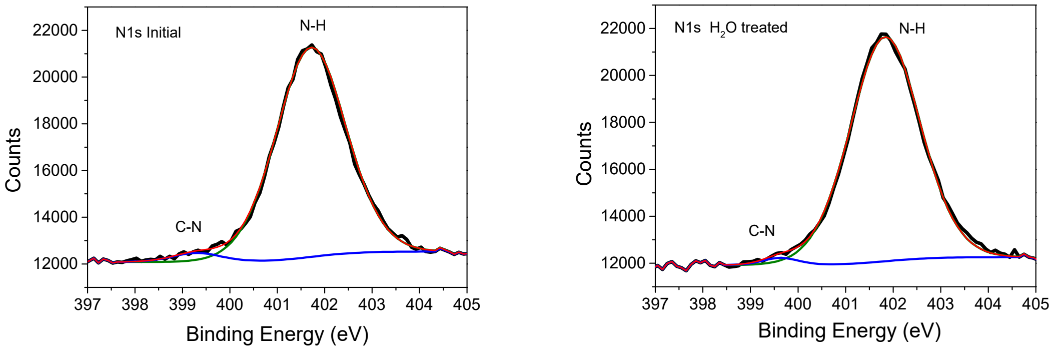

Figure S4. XPS analysis on $MA_2CuCl_2Br_2$ of N1s binding energy measured in a fresh sample (left), and after moisture exposure (right).

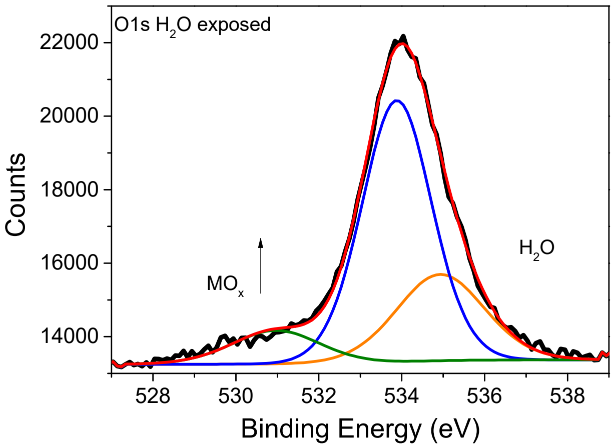

Figure S5. XPS analysis on $MA_2CuCl_2Br_2$ of O1s binding energy measured after moisture exposure.

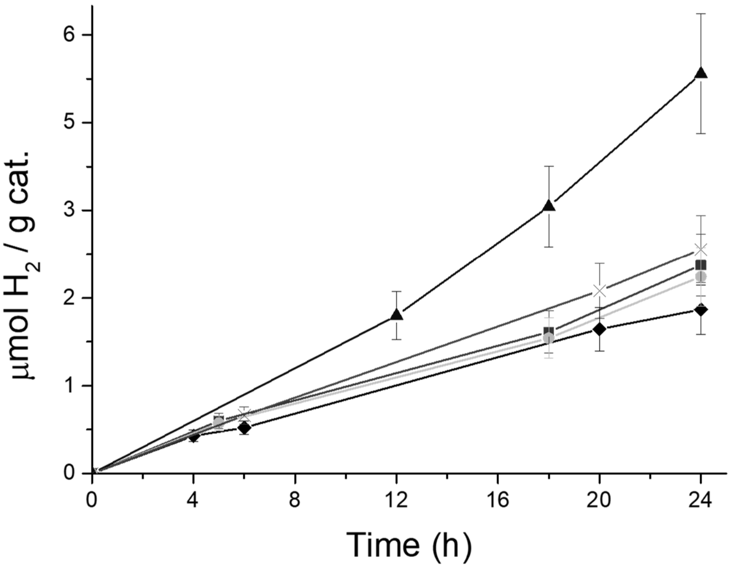

Figure S6. H_2 generation upon UV-Vis irradiation of hybrid lead halide perovskites: a) $MAPbBrI_2$, b) $MAPbClI_2$, c) $MAPbI_3$, d) $MAPbCl_3$ and e) $MAPbBr_3$ as function of time. Reaction conditions: 10 mg of photocatalyst as thick bed and irradiation with a solar simulator (1000 W/m^2).

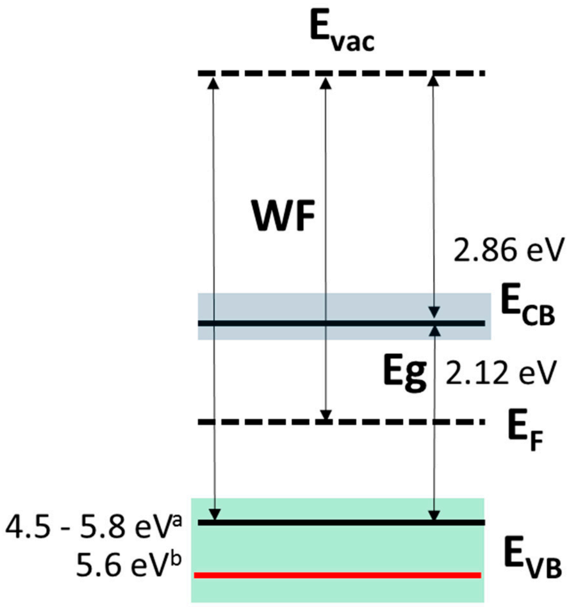

Scheme S1. Energy Level position based on the UPS measurements of the reference copper perovskite. [a]Based on data reported in reference 36, [b]based in our XPS measurements.

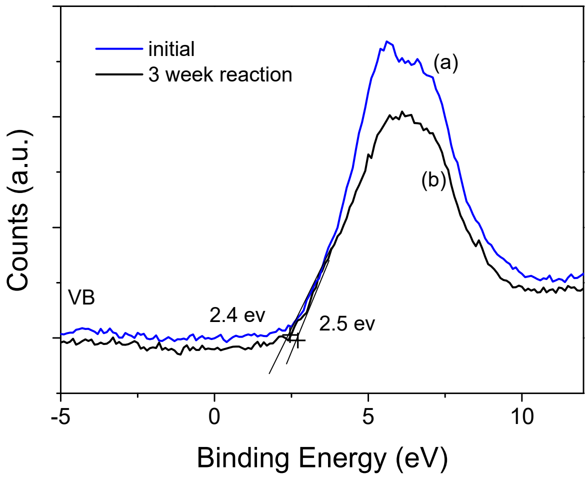

Figure S7. VB determination by XPS analysis of fresh $MA_2CuCl_2Br_2$ (a) and after moisture exposure (b).

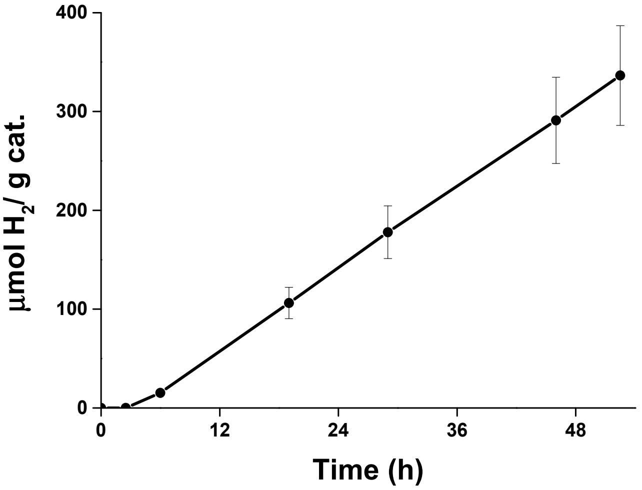

Figure S8. Temporal evolution of H_2 generation upon sunlight irradiation of H_2O vapors in the presence of hybrid copper halide perovskite $MA_2CuCl_2Br_2$.

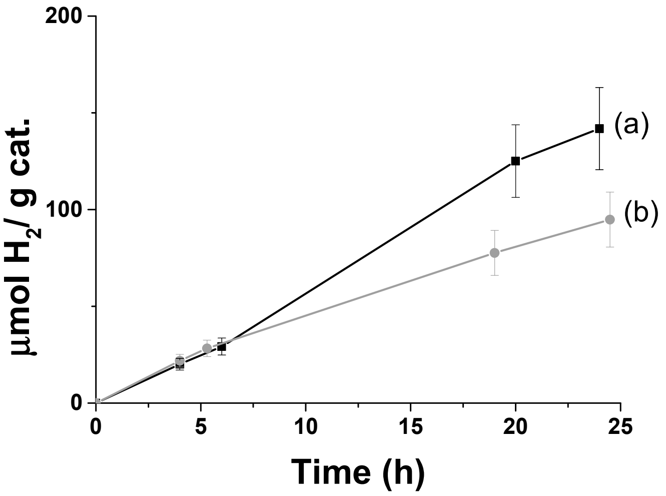

Figure S9. Temporal evolution of H_2 generation upon sunlight irradiation of H_2O vapors in the presence of hybrid copper halide perovskite $MA_2CuCl_2Br_2$ (a) and $MA_2CuCl_{0.5}Br_{3.5}$ (b).

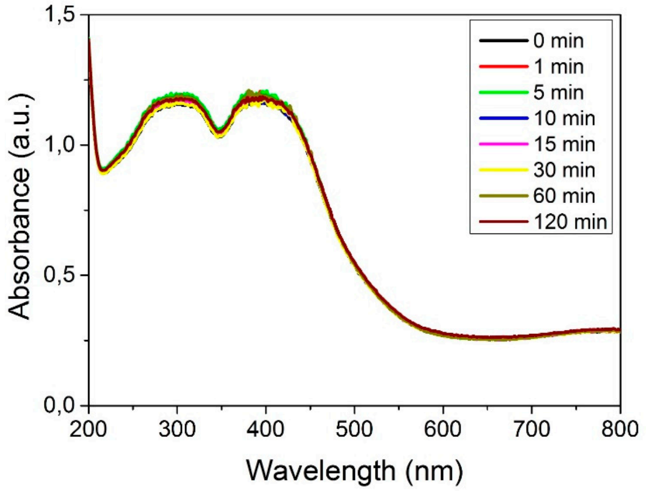

Figure S10. Optical absorption spectrum of the $MA_2CuCl_2Br_2$ film as a function of laser time irradiation.
